# Smurf2 Suppresses Proliferation and Cell Cycle of Triple‐Negative Breast Cancer Cells by Promoting the Polyubiquitination and Degradation of RPL35A

**DOI:** 10.1111/jcmm.70394

**Published:** 2025-01-27

**Authors:** Siyu Wei, Yuying Liu, Zhihao Wang, Ti Wei, Wenkai Zhou, Wanwan Li, Jiaxin Zhang, Zhiyi Liu, Zhao Liu

**Affiliations:** ^1^ Department of General Surgery The Affiliated Hospital of Xuzhou Medical University Xuzhou Jiangsu China; ^2^ Institute of Digestive Diseases Xuzhou Medical University Xuzhou Jiangsu China; ^3^ Research Center of Digestive Diseases The Affiliated Hospital of Xuzhou Medical University Xuzhou Jiangsu China

**Keywords:** L35a ribosomal protein, proliferation and cell cycle, SMAD‐specific E3 ubiquitin protein ligase 2, triple‐negative breast cancer, ubiquitination

## Abstract

Human L35a ribosomal protein (RPL35A) has been reported to confer higher drug resistance and viability to triple‐negative breast cancer (TNBC) cells, but the mechanism related to its promotion of TNBC malignant progression is still unclear. Here, we found that silencing of RPL35A could inhibit the proliferation of TNBC cells by suppressing the G1/S phase transition. Furthermore, SMAD‐specific E3 ubiquitin protein ligase 2 (Smurf2) was found to be a potential upstream ubiquitin ligase of RPL35A. Smurf2 could interact with RPL35A and promote its degradation and K63‐linked polyubiquitination, thereby suppressing the G1/S phase transition and proliferation of TNBC cells. In addition, the roles of Smurf2 were confirmed in a xenograft mouse model. Finally, we found a negative correlation between the protein levels of RPL35A and Smurf2 in human TNBC tissues. In summary, Smurf2 inhibits the proliferation of TNBC cells by blocking the cell cycle process, which is associated with regulating RPL35A.

## 1 Introduction

Breast cancer poses a substantial health challenge for women, attributable to its high mortality and morbidity rates [1]. Recent data indicate that breast cancer is the most commonly diagnosed cancer in women, second only to lung cancer in mortality [2]. It has been reported that histological subtypes are associated with prognosis [3, 4]. Patients with triple‐negative breast cancer (TNBC) are insensitive to radiotherapy, endocrine and targeted therapies, except for surgery, due to the lack of oestrogen receptor (ER), progesterone receptor (PR) and human epidermal growth factor receptor (HER2). Therefore, it is urgent to find new therapeutic approaches for TNBC.

Human L35a ribosomal protein (RPL35A) is a ribosomal protein consisting of 110 amino acids located at chromosomal region 3q29‐qter, essential for encoding the protein component of the 60S large ribosomal subunit [5, 6], and is required for large ribosomal subunit assembly and rRNA pre‐processing. It has been reported that RPL35A is mainly involved in the development of Diamond‐Blackfan anaemia [7, 8] and acts as a biomarker in tumour angiogenesis [9]. Recent studies show that RPL35A is significantly upregulated in various human tumours and closely linked to tumorigenesis and malignant progression. For example, silencing of RPL35A can inhibit the malignant behaviour of gastric cancer cells by regulating the expression level of the phosphorylated kinase [10]; LncRNA NB1 binding to RPL35A can promote neuroblastoma development and metastasis [11]; up‐regulation of RPL35A can reduce the sensitivity of cancer cells to radiotherapy and chemotherapy [12]; silencing of RPL35A can inhibit the progression of ovarian cancer by inhibiting the binding of YY1 and CTCF promoter [13]. RPL35A may serve as a promising biomarker for various human cancers, but its specific role and mechanisms in triple‐negative breast cancer (TNBC) remain unclear and require further investigation.

SMAD‐specific E3 ubiquitin protein ligase 2 (Smurf2), a member of the HECT‐type NEDD4 E3 ligase family, is crucial for identifying substrates destined for ubiquitination and proteasomal degradation [14]. Smurf2 consists of an N‐terminal C2 structural domain, three WW structural domains, and an evolutionarily conserved C‐terminal HECT structural domain [15], and is involved in a variety of biological processes, including tumour metastasis, apoptosis, proliferation, cell cycle progression and genome stability [16, 17]. The dysregulated expression of Smurf2 has been reported across a wide spectrum of human tumours, such as colorectal carcinoma, pancreatic cancer, lung cancer and hepatocellular carcinomas [18, 19, 20]. In addition, Smurf2 protein expression is significantly down‐regulated in invasive breast cancer and correlates with the malignant progression of triple‐negative breast cancer (TNBC). Silencing Smurf2 may reduce the tumorigenic properties of human breast cancer cells by lowering CNKSR2 expression levels [21, 22]. Furthermore, Smurf2 is also able to reduce the breast cancer cell migration and inhibit bone metastasis by degrading Smurf1 protein [23]. However, the role of Smurf2 as an ubiquitin ligase in breast cancer, especially TNBC, remains to be further verified.

This study establishes that RPL35A promotes tumour growth in triple‐negative breast cancer (TNBC). Our findings show that knocking down RPL35A significantly inhibits TNBC cell proliferation by affecting the cell cycle. We also found that upregulation of RPL35A is associated with the ubiquitin‐proteasome system (UPS). Evidence indicates that Smurf2 enhances K63‐linked polyubiquitination and degradation of RPL35A, thereby inhibiting TNBC cell growth. Additionally, we clarified the correlation between RPL35A and Smurf2 expression in clinical samples.

## 2 Materials and Methods

### 2.1 Antibodies and Plasmids

Smurf2 (Cat No. A2278) antibody was bought from Abclonal (Wuhan, Hubei, China). CyclinD1 (Cat No. 60186‐1‐Ig), CyclinE2 (Cat No. 11935‐1‐AP), RPL35A (Lot: GR3259679‐3), 3 × Flag (Cat No. 66008‐4‐Ig), HA (Cat No. 51064‐2‐AP) and Myc (Cat No. 60003‐2‐Ig) antibodies were purchased from Proteintech (Wuhan, Hubei, China). GAPDH (Cat No. M1310‐2) antibodies were bought from Huabio (Hangzhou, Zhejiang, China). Myc‐SMURF2, 3 × Flag‐RPL35A, Myc‐Vector, 3 × Flag‐Vector and HA‐Ub (WT, K48R and K63R) plasmids were purchased from Youbio (Changsha, Hunan, China).

### 2.2 Tissue Samples

Breast cancer samples and corresponding paracancerous tissue samples were provided by the Affiliated Hospital of Xuzhou Medical University. All tissue samples were obtained from patients who underwent unilateral radical mastectomy for breast cancer from December 2021 to March 2022. The isolated tissue specimens were labelled and immediately frozen in liquid nitrogen and stored at −80°C. The study was approved by the Ethics Committee of The Affiliated Hospital of Xuzhou Medical University (approval no. XYFY2022‐KL405‐01). Informed consent was obtained from patients for all samples.

### 2.3 Cell Culture

293T, MDA‐MB‐231 and SUM149PT cell lines were purchased from Stem Cell Bank, Chinese Academy of Sciences (Shanghai, China) in Jun. 2023. Each cell line was passaged in our laboratory for fewer than 6 months after receipt or resuscitation. The STR results of 293T are as follows: D5S818: 8,9; D13S317: 12,14; D7S820: 11; D16S539: 9,13; vWA: 16,19; THO1: 7,9.3; Amelogenin: X; TPOX: 11; CSF1PO: 11,12. The STR results of MDA‐MB‐231 are as follows: Amelogenin: X; CSF1PO: 12,13; D13S317: 13; D16S539: 12; D5S818: 12; D7S820: 8,9; THO1: 7,9.3; TPOX: 8,9; vWA: 15,18. The STR results of SUM149PT are as follows: CSF1PO: 12, 12; D13S317: 12, 12; D16S539: 11, 11; D5S818: 11, 11; D7S820: 11, 11; THO1: 9.3, 9.3; TPOX: 9, 9; vWA: 16, 18; Amelogenin: X, X. The cells were cultured in DMEM (293T, SUM149PT) and L‐15 (MDA‐MB‐231) (Yuanpei, Shanghai, China) supplemented with 10% fetal bovine serum (FBS, Gibco, Shanghai, China) at 5% CO_2_ at 37°C.

### 2.4 Plasmid Construction

Two lentiviral short hairpin RNA (shRNA) lentiviral vectors against RPL35A were constructed by Hunan Youbao Company, China, with the following target sequences: the forward primer of shRPL35A#1 was 5′‐GGGCAAGAGAUGCGCUUAUTT‐3′, and the reverse primer was 3′‐AUAAG‐CGCAUCUCUUGCCCTT‐5′; the forward primer of shRPL35A#2 was 5′‐CCCAUGGAAACAGUG‐GCAUTT‐3′, and the reverse primer was 3′‐AUGCCACUGUUUCCAUGGGTT‐5′. It was subcloned into the pLV‐shRNA lentiviral vector.

### 2.5 Cell Cycle Analysis

The treated cells were collected at the designated time, added 500 μL of 70% cold ethanol for fixation, and fixed at 4°C overnight. The cells were reselected with PBS at room temperature to wash off the fixative, added 500 μL of pre‐configured staining working solution (Rnase: PI = 1:9), and reacted for 30–60 min at room temperature, protected from light, and then analysed the DNA content of the cells using CytoFLEX flow cytometer.

### 2.6 Cell Viability Assay

Cell viability was assayed by a cell counting kit (CCK‐8, ApexBio, China). Briefly, cells were inoculated into 96‐well plates at a density of 8000 cells/200 μL/well and repeated 5 times. CCK‐8 reagent 10 μL/well was added at the indicated time. After incubation at 37°C and protected from light for 4 h, the absorbance at 450 nm was detected using a SynergyMx multimode enzyme marker. The cell viability was calculated based on the absorbance value.

### 2.7 Colony Formation Assay

700 cells were inoculated in 6‐well plates and cultured continuously until clones were visible. The cells were then fixed with 4% paraformaldehyde and stained with 0.3% crystal violet. After washing with PBS, the plates were photographed with a digital camera. Colony formation was confirmed by manual counting, with colony size defined as > 50 cells.

### 2.8 Western Blotting

Cells were lysed in RIPA buffer, and the supernatant was collected by centrifugation at 12,000× g and 4°C for 10 min. Aliquots were processed by SDS‐PAGE and transferred to PVDF membranes, which were closed with BSA solution. The membrane was incubated with the primary antibody overnight at 4°C, washed, and incubated with the secondary antibody at room temperature for 1 h. Protein bands were detected using ECL Plus protein blotting substrate (Thermo Fisher Scientific, Waltham, MA, USA) and chemiluminescent detection system (Tanon, Shanghai, China) for Visualisation. Band density was quantified using ImageJ software (National Institutes of Health, Bethesda, MD, USA). Relative protein levels were determined by normalising the optical density values of target proteins to GAPDH.

### 2.9 Co‐Immunoprecipitation

Cells were lysed in 500 μL of chilled IP buffer (1% Triton‐X‐100, 150 mM NaCl, 20 mM HEPES, 2 mM EDTA, 5 mM MgCl_2_, pH 7.4), and the supernatant was incubated with the indicated antibodies overnight at 4°C, followed by co‐incubation with the magnetic beads for 4 h. The magnetic beads were washed three times with 1 × TBS, boiled in 2 × loading buffer for 10 min and the immunoprecipitates were analysed by western blotting.

### 2.10 Ubiquitination Assays

293T and TNBC cells were successfully transfected with the desired plasmids and 500 μL of frozen IP buffer lysate was added to the cells. Total cell lysates were first incubated with the indicated antibodies overnight, and then the magnetic beads were co‐incubated for 4 h. Samples were washed three times with TBS and collected for western blotting analysis.

### 2.11 Immunohistochemistry

Immunohistochemistry (IHC) analysis was performed using a kit (Zhongshan Goldenbridge Biotech, Beijing, China) according to manufacturer instructions. Tissue specimens were collected, fixed in 4% paraformaldehyde, sectioned in paraffin, blocked with 10% goat serum, and incubated at room temperature with primary antibodies followed by secondary antibodies conjugated with horseradish peroxidase. After incubation in 3,3′‐diaminobenzidine solution, the sections were counterstained with haematoxylin, dehydrated, and sealed. Images were obtained using a microscope and analysed using Image‐Pro‐Plus software (v.6.0; Media Cybernetics, Bethesda, MD, USA). Protein expression was represented as the mean optical density.

### 2.12 RNA Sequencing

We sent the cells to UW Bio for RNA sequencing, and the sequencing protocol was divided into two groups for two‐by‐two comparisons, with each group including three samples. One group was the control group, MDA‐MB‐231 cells transferred to shControl; the other group was the experimental group, three dishes of MDA‐MB‐231 cells with knockdown of RPL35A. After sequencing, clean reads were compared to the reference gene sequences using Bowtie2, and then RSEM was used to calculate the gene expression levels of each sample, and differential genes (DEGs) between the two groups were searched based on the differences in gene expression levels.

### 2.13 Tumour Xenografts in Nude Mice

Twelve BALB/c female nude mice (4 weeks old) were housed under specific pathogen‐free conditions with free access to food and water. Myc‐Vector or Myc‐Smurf2 MDA‐MB‐231 cells (1 × 10^6^ cells per mouse) were injected into the right subcutaneous fat pad of mice. Mice were monitored and were measured by a slide calliper when a macroscopic tumour appeared. Tumour volume (mm^3^) = π/6 × length×width^2^. Thereafter, tumour size was measured every 2 days to calculate its volume. At day 52, these mice were sacrificed and tumour volume and weight were measured.

### 2.14 Public Database Analysis

RPL35A expression in TNBC and normal breast tissue was obtained from Kaplan–Meier Plotter (http://www.kmplot.com/analysis/). We chose the basal classification in Subtype STGallen, where the number of TNBC samples is 846 and the evaluation metric is RFS. The potential upstream ligase of RPL35A were anticipated using Ubibrower (http://ubibrowser.bio‐it.cn/ubibrowser/). The potential interaction molecules of RPL35A were predicted by GeneCards (https://www.genecards.org/).

### 2.15 Statistical Analysis

In our study, at least three independent experiments were conducted for each method and the results of one representative experiment were shown. All quantitative data were expressed as mean ± SD. Comparisons of normality, chi‐square, and independent samples were performed using *t*‐tests, and differences between multiple groups were determined by one‐way analysis of variance (ANOVA) followed by post hoc tests. Statistical analyses were performed using SPSS 22.0 software. GraphPad Prism9 software was used to draw statistical graphs. Differences were considered statistically significant at *p* < 0.05.

## 3 Results

### 3.1 Knockdown of RPL35A Inhibits the Proliferation of TNBC Cells

**FIGURE 1:**
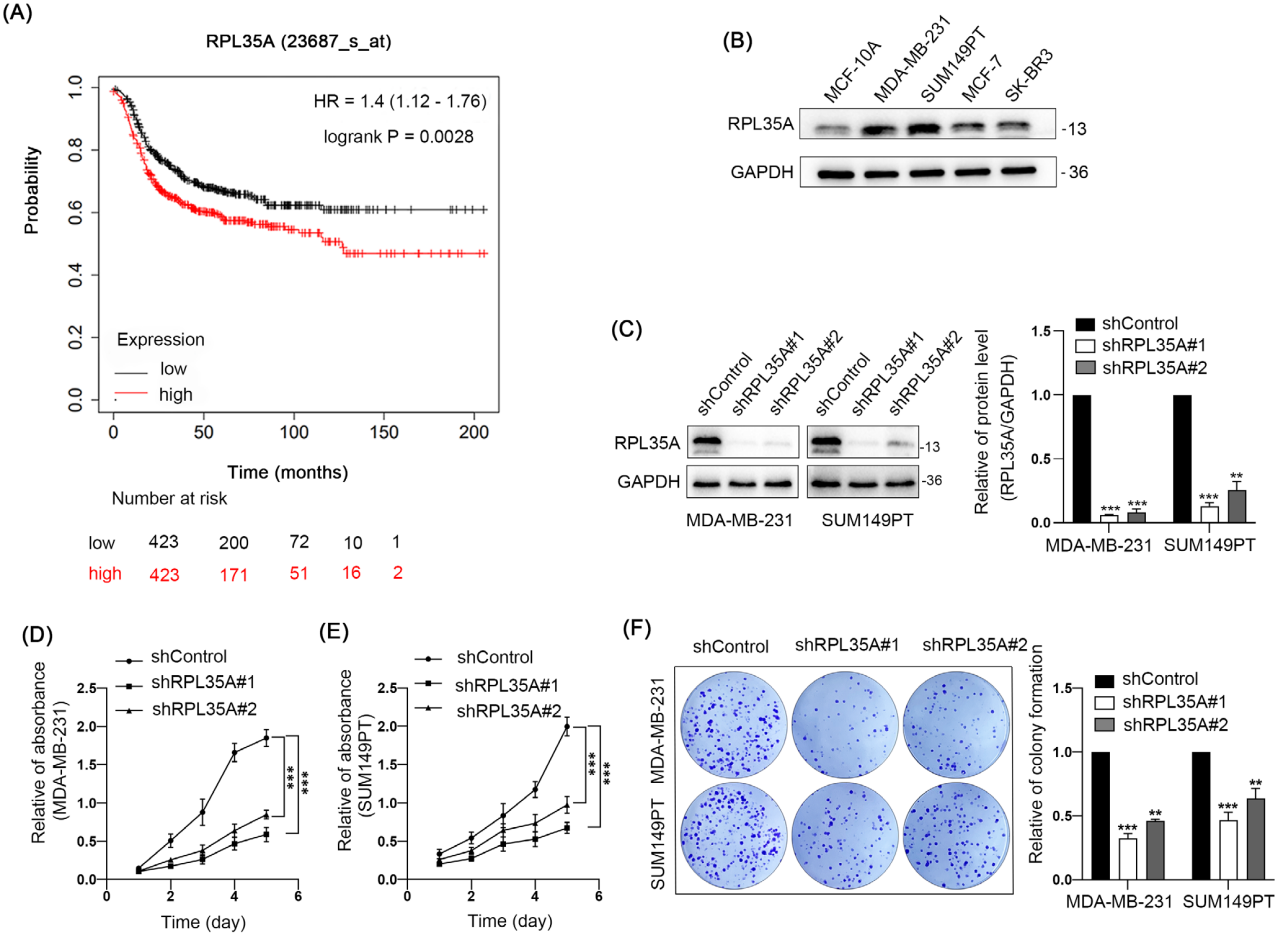
Knockdown of RPL35A inhibits the proliferation of TNBC cells. (A) Correlation of high RPL35A expression with reduced overall survival in TNBC patients (hazard ratio = 1.4, 95% confidence interval [CI] = 1.12 to 1.76, *p* = 0.0028). (B) Representative blots of RPL35A protein level in different cell lines. (C) Representative blots and quantification efficiency of shRPL35A. (D and E) CCK8 assay results. (F) Representative images and quantitative analyses of RPL35A‐silenced MDA‐MB‐231 and SUM149PT clone formation experiments. **p < 0.01, ***p < 0.001.

To clarify RPL35A's role in TNBC progression, we first examined the correlation between RPL35A expression levels and patient survival outcomes using data from TCGA. It was found that high expression of RPL35A was associated with poor prognosis (Figure 1A). We detected the protein level of RPL35A on numerous breast cancer cell lines using western blotting and ascertained that the expression level of RPL35A was the utmost in two TNBC cell lines, namely MDA‐MB‐231 and SUM149PT (Figure 1B). Then, we performed loss‐of‐function experiments to verify the role of RPL35A in cell proliferation using MDA‐MB‐231 and SUM149PT. First, two specific shRNAs (shRPL35A#1, shRPL35A#2) were selected to down‐regulate RPL35A, and the knockdown efficiency of shRNAs was verified by western blotting (Figure 1C). Next, cell growth was determined using TNBC cells silenced RPL35A. CCK‐8 assays showed that knockdown of RPL35A obviously attenuated the cell viability of MDA‐MB‐231 and SUM149PT (Figure 1D,E). In addition, silencing of RPL35A significantly inhibited the colony‐forming ability of TNBC cells, compared with the control group (Figure 1F). These results indicate that silencing of RPL35A inhibits the proliferation of TNBC cells.

### 3.2 Knockdown of RPL35A Suppresses G1/S Phase Transition of TNBC Cells

**FIGURE 2:**
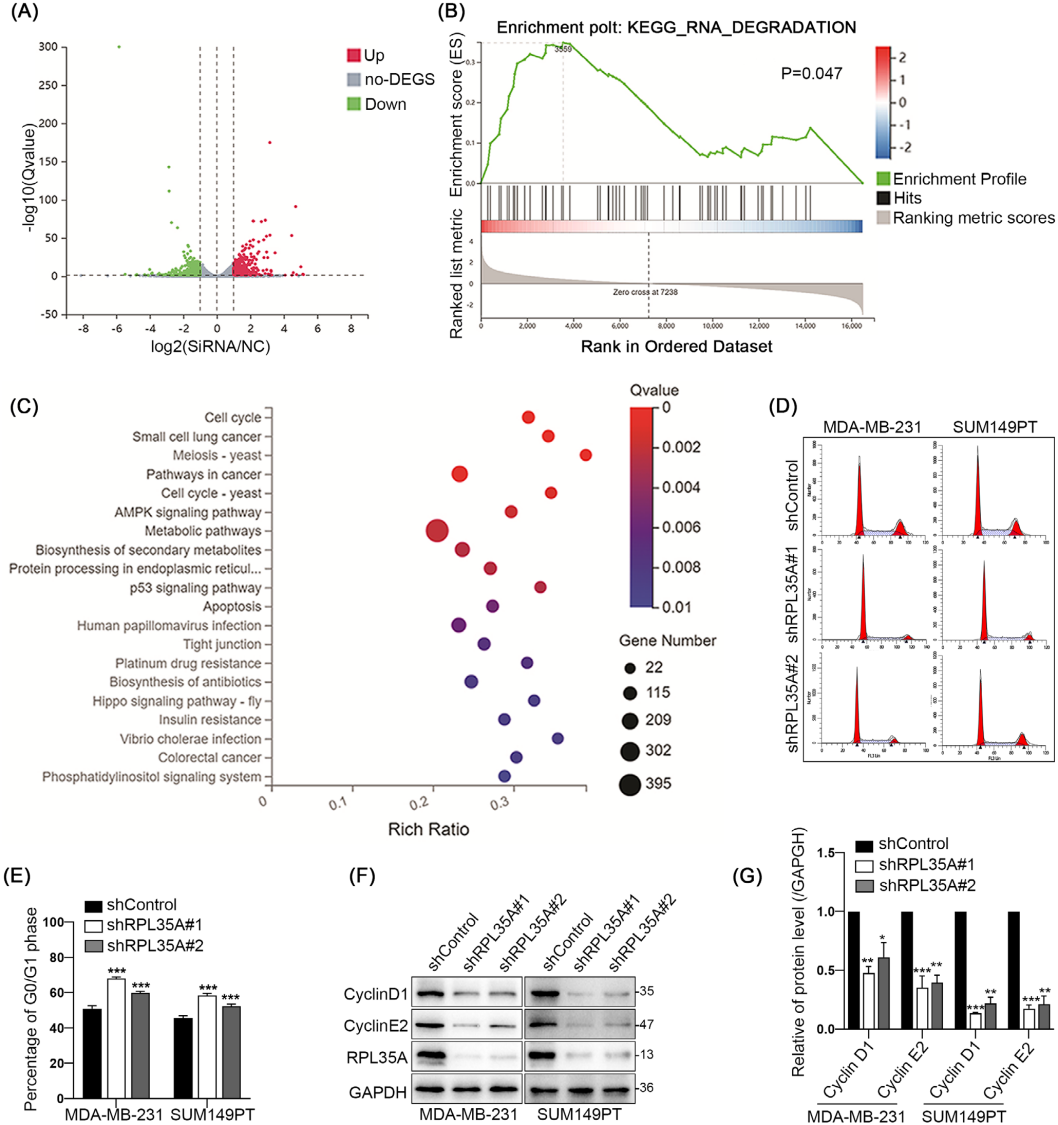
Knockdown of RPL35A suppresses G1/S phase transition of TNBC cells. (A) Volcano diagram. (B and C) GSEA and KEGG analyses revealed that the differential genes were mainly enriched in relevant pathways and functions such as cell cycle. (D and E) Quantitative analysis of G0/G1 phase cell ratio after RPL35A silencing. (F and G) Representative blots and quantitative analysis showing protein levels of CyclinD1 and CyclinE2 in RPL35A‐silenced MDA‐MB‐231 and SUM149PT cells. *p < 0.05, **p < 0.01, ***p < 0.001.

To clarify how RPL35A promotes the proliferation of TNBC cells, we conducted RNA sequencing on MDA‐MB‐231 cells with RPL35A knockdown. A total of 4244 differentially expressed genes were identified, comprising 2192 upregulated and 2052 downregulated genes (Figure 2A). The differential genes were found to be mainly enriched in cell cycle, P53 signalling pathway, RNA degradation, small cell lung cancer, and AMPK signalling pathway by GSEA and KEGG analysis (Figure 2B,C). To assess the impact of RPL35A silencing on TNBC cells during different cell cycle phases, we conducted flow cytometry assays for both shControl and shRPL35A groups. It was found that knockdown of RPL35A significantly increased the percentage of cells in the G0/G1 phase and decreased the distribution of S and G2 phase cells, indicating that down‐regulation of RPL35A inhibited the G1/S phase transition of the cell cycle (Figure 2D,E). Next, we assessed the expression of key regulators associated with the G1 phase, such as Cyclin D1 and Cyclin E2. The results showed that knockdown of RPL35A obviously decreased the expression of CyclinD1 and Cyclin E2 (Figure 2F,G). These results indicate that knockdown of RPL35A suppresses the G1/S phase transition of TNBC cells.

### 3.3 Smurf2 Destabilises RPL35A Through Promoting Its K63‐Linked Polyubiquitination and Degradation via the Proteasome Pathway

**FIGURE 3:**
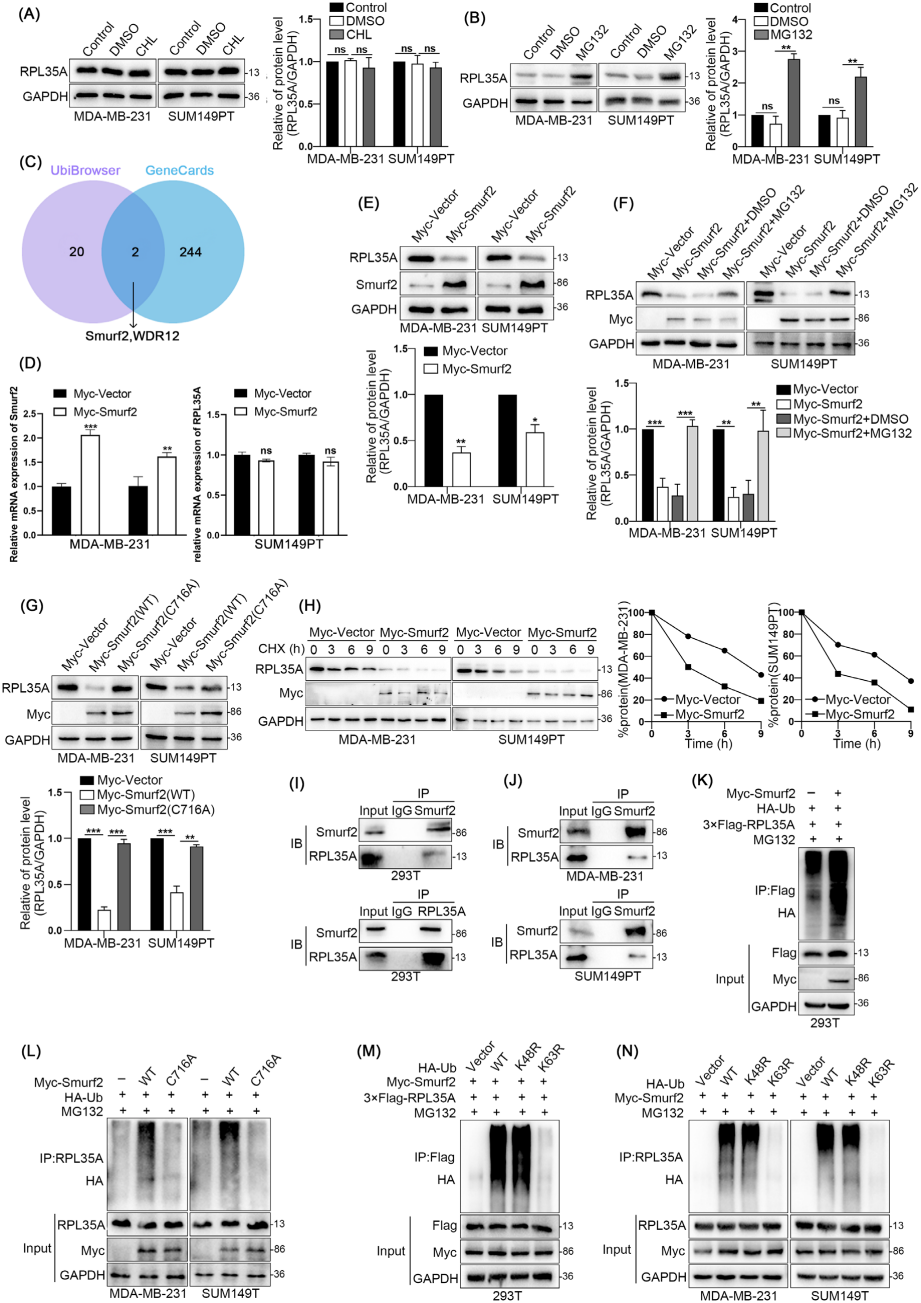
Smurf2 destabilises RPL35A through promoting its K63‐linked polyubiquitination and degradation via the proteasome. (A and B) Representative blots and quantitative analysis showing the protein level of RPL35A in cells after CHL and MG132 treatment. (C) Smurf2 was predicted to be the most likely ubiquitin ligase for RPL35A by UbiBrowser and GeneCards databases. (D) Quantitative analysis showing mRNA levels of RPL35A in MDA‐MB‐231 and SUM149PT cells overexpressing Smurf2. (E) Representative blots and quantitative analysis showing protein levels of RPL35A in MDA‐MB‐231 and SUM149PT cells overexpressing Smurf2. (F) Representative blots and quantitative assays showed that MG132 blocked RPL35A degradation in Smurf2 upregulated cells. (G) Representative blot and quantitative analysis of RPL35A expression in BC cells with Smurf2 mutations. (H) Representative blots and quantitative analysis showed that overexpression of Smurf2 reduced the stability of RPL35A. (I and J) Immunoprecipitation experiments show that Myc‐tagged Smurf2 interacts with Flag‐tagged RPL35A. (K) Representative blots of Smurf2 ubiquitinated RPL35A in 293 T. (L) Representative blots of ubiquitinated RPL35A in TNBC cells with Smurf2 mutations. (M and N) Smurf2 ubiquitinate RPL35A in TNBC cells via Lys63‐linked ubiquitin chains. *p < 0.05, **p < 0.01, ***p < 0.001.

Numerous studies have highlighted the crucial role of the proteasome pathway in regulating protein stability [24]. To investigate the degradation mode of RPL35A, we treated TNBC cells using proteasome inhibitor MG132 and lysosome inhibitor chloroquine (CHL) respectively. It was found that MG132 significantly inhibited RPL35A protein degradation, while CHL had no effect (Figure 3A,B). Recent reports indicate that E3 ubiquitin ligase is essential to the UPS, the main mechanism regulating protein stability [25]. To identify the upstream ubiquitin ligase modulating RPL35A, we used the UbiBrowser and GeneCards databases to screen for potential ligases and the interacting proteins. A Venn diagram revealed two candidate targets, with WDR12 identified as a non‐classical E3 ubiquitin ligase (Figure 3C). Thus, we speculated that Smurf2 might be the potential upstream E3 ubiquitin ligase of RPL35A. Next, we verified the regulatory of Smurf2 on RPL35A expression. MDA‐MB‐231 and SUM149PT cells were used to establish stable cell lines overexpressed Smurf2 (Myc‐tag). It was found that overexpression of Smurf2 obviously decreased the RPL35A protein level but not mRNA (Figure 3D,E). Then we treated TNBC cells overexpressed Smurf2 using MG132 to determine whether Smurf2‐induced RPL35A degradation is dependent on the proteasome system. The results showed that MG132 significantly restored the reduced expression of RPL35A caused by Smurf2 overexpression (Figure 3F). To evaluate the role of Smurf2's E3 ligase activity in RPL35A degradation, we created a Smurf2‐HECT mutant by substituting Cys716 in the HECT domain with alanine (C716A). Previous studies revealed that this mutant has no E3 ligase activity [26, 27]. Notably, Smurf2/C716A lost the ability to decrease RPL35A protein level, compared to wild‐type Smurf2 (Smurf2‐WT) (Figure 3G). We subsequently evaluated the influence of Smurf2 on the protein stability of RPL35A. TNBC cells overexpressing Smurf2 were treated with the protein synthesis inhibitor cycloheximide (CHX) at different times. It was found that, in TNBC cells with high Smurf2 expression, the half‐life of endogenous RPL35A protein was significantly shorter than in the control group (Figure 3H).

The results above suggest that Smurf2 destabilises RPL35A through the proteasome pathway, relying on its ubiquitin ligase activity. It has been reported that Smurf2 could act as a ubiquitin ligase to promote the ubiquitination of several substrates [28]. To elucidate the mechanism by which Smurf2 modulates RPL35A, we first investigated their interaction using a co‐immunoprecipitation assay. It was found that Smurf2 could interact with endogenous RPL35A both in 293 T and TNBC cells (Figure 3I,J). Subsequently, we performed an intracellular ubiquitination assay to elucidate the effect of Smurf2 on the ubiquitination of RPL35A. Compared with the control group, cells overexpressed Smurf2 exhibited significantly increased ubiquitination levels of 3 × Flag‐RPL35A in 293 T cells (Figure 3K). Correspondingly, the Smurf2‐HECT mutant markedly reduced the ability of Smurf2 promoting the ubiquitination of endogenous RPL35A in TNBC cells (Figure 3L).

The ubiquitination process within the proteasome pathway is mediated by seven lysine residues, with K48 and K63 representing the predominant types. To further clarify the mechanism Smurf2 promoting the ubiquitination of RPL35A, we co‐transfected wild‐type or mutant HA‐Ub as well as Myc‐Smurf2 and/no 3 × Flag‐RPL35A into cells and analysed the ubiquitination level of exogenous/endogenous RPL35A by western blotting. It was obvious that the polyubiquitination level of 3 × Flag‐RPL35A was initiated by the K63‐linked Ub‐chain in 293T cell (Figure 3M), as well as endogenous RPL35A in TNBC cells (Figure 3N). In conclusion, these results suggest that Smurf2 destabilises RPL35A by promoting its K63‐linked polyubiquitination and degradation via the proteasome pathway.

### 3.4 Smurf2 Inhibits G1/S Phase Transition and Proliferation of TNBC Cells Through Regulating RPL35A

**FIGURE 4:**
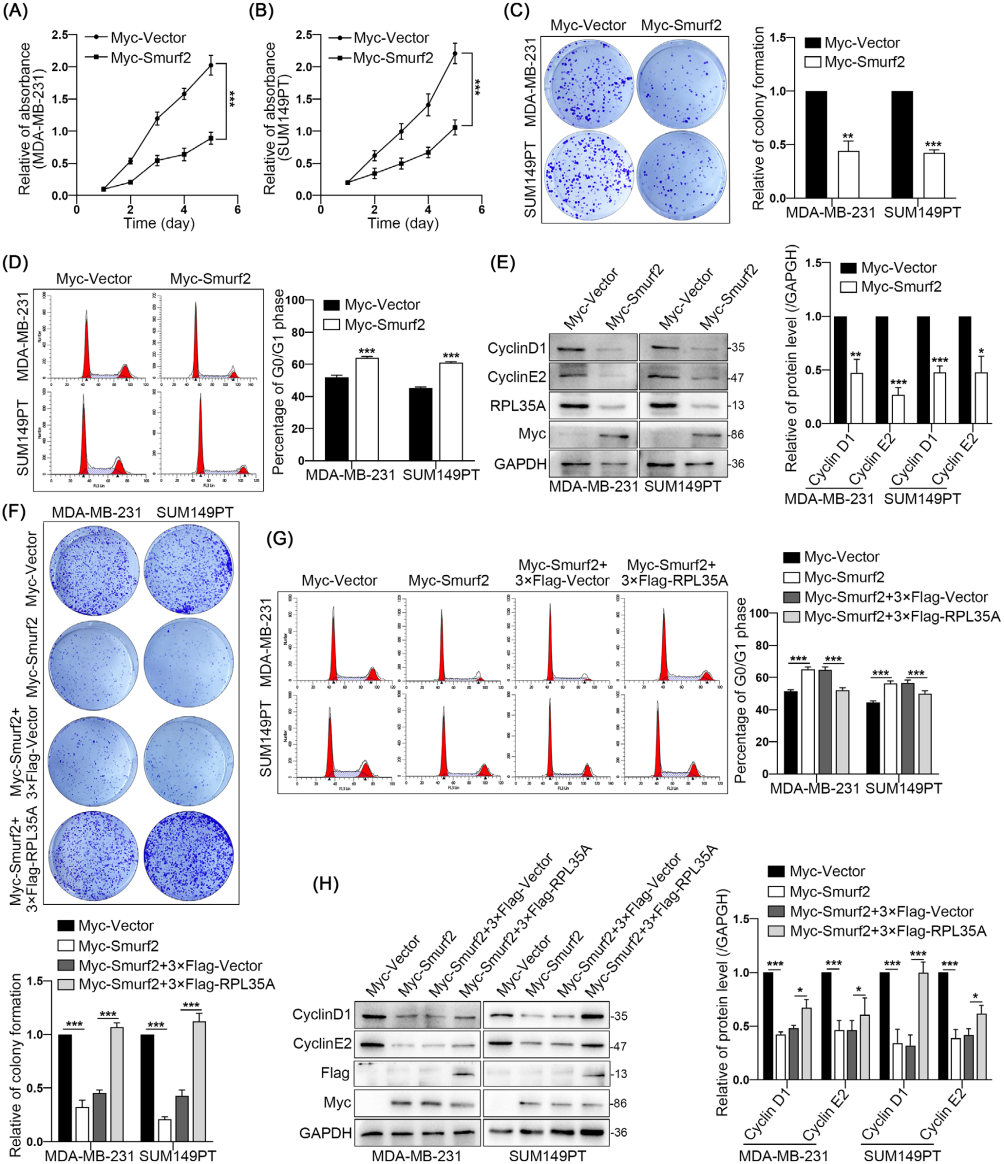
Smurf2 inhibits G1/S phase transition and proliferation of TNBC cells through regulating RPL35A. (A and B) CCK8 assay results. (C) Representative images and quantitative analysis of MDA‐MB‐231 and SUM149PT clone formation assays overexpressing Smurf2. (D) Quantitative analysis of G0/G1 phase cell ratio after overexpression of Smurf2. (E) Representative blots and quantitative analysis showing protein levels of CyclinD1 and CyclinE2 in MDA‐MB‐231 and SUM149PT cells overexpressing Smurf2. (F) Quantitative analysis showed that overexpression of RPL35A effectively reversed the inhibition of clone formation caused by overexpression of Smurf2. (G) Quantitative analysis showed that overexpression of RPL35A effectively reduced the ratio of G0/G1 phase cells. (H) Representative blots and quantitative analysis showed that overexpression of RPL35A effectively upregulated the protein levels of CyclinD1 and CyclinE2 in cells. *p < 0.05, **p < 0.01, ***p < 0.001.

The findings presented above suggest that Smurf2 effectively downregulates the protein expression level of RPL35A. To further investigate the effect of Smurf2 on TNBC cell proliferation and cell cycle, we performed a functional acquisition assay and found that overexpression of Smurf2 significantly inhibited cell viability (Figures 4A,B) and colony formation (Figure 4C). Correspondingly, Smurf2 overexpression similarly suppressed the G1/S phase transition in the cell cycle (Figures 4D) and inhibited the protein expression of CyclinD1 and CyclinE2 (Figure 4E). To determine whether Smurf2 inhibits the proliferation and G1/S phase transition of TNBC cells via the modulation of RPL35A, we performed a rescue experiment by overexpressing 3 × Flag‐RPL35A in cells exhibiting elevated levels of Smurf2. It was found that overexpression of RPL35A effectively reversed the inhibitory effect of Smurf2 on cell proliferation (Figure 4F) and the proportion of cells in the G0/G1 phase of the cell cycle (Figure 4G), and also upregulated the expression of CyclinD1 and CyclinE2 (Figure 4H). Taken together, these results suggest that Smurf2 inhibits G1/S phase transition and proliferation of TNBC cells through regulating RPL35A.

### 3.5 Smurf2 Inhibits Tumour Growth In Vivo

**FIGURE 5:**
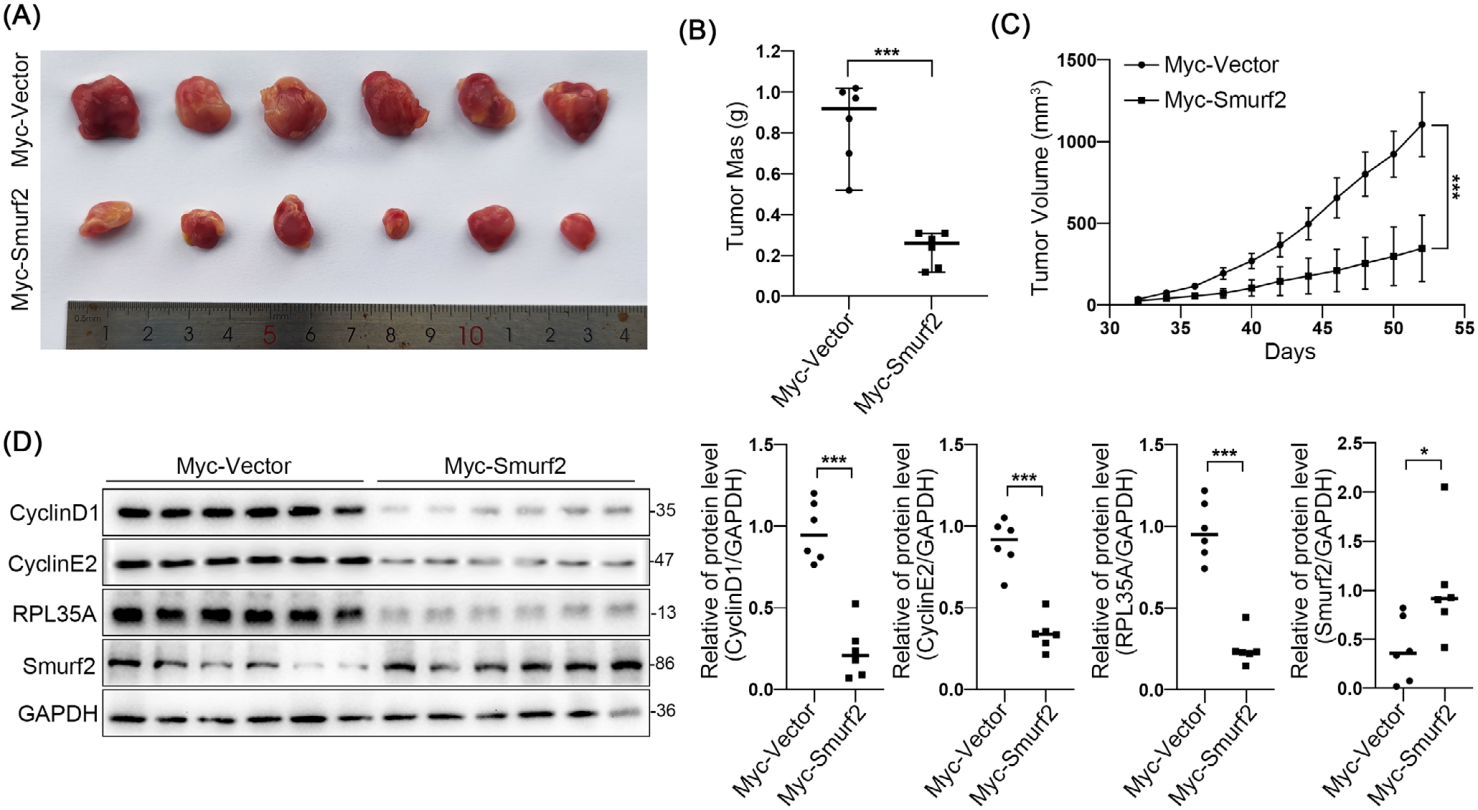
Smurf2 inhibits TNBC cell growth by regulating RPL35A in vivo. (A) Images of tumour in nude mice. (B) Tumour weight of nude mice. (C) Tumour volume (every 2 days) of nude mice. (D) Protein levels of RPL35A, CyclinD1 and CyclinE2 in xenograft tissues. ***p < 0.001.

To verify the effect of Smurf2 on tumorigenesis and development in vivo, we performed mouse xenograft model by injecting MDA‐MB‐231 cells overexpressing Smurf2 into BALB/c nude mice (Figure 5A). We found that high expression of Smurf2 resulted in reduced tumour volume and weight (Figure 5B,C). Moreover, western blotting revealed that, compared to the control group, RPL35A protein was significantly decreased in tumours with upregulation of Smurf2 (Figure 5D). These results suggest that Smurf2 can inhibit tumour growth by regulating RPL35A in vivo.

### 3.6 Smurf2 Expression Is Inversely Correlated With RPL35A in Human TNBC Tissues

**FIGURE 6:**
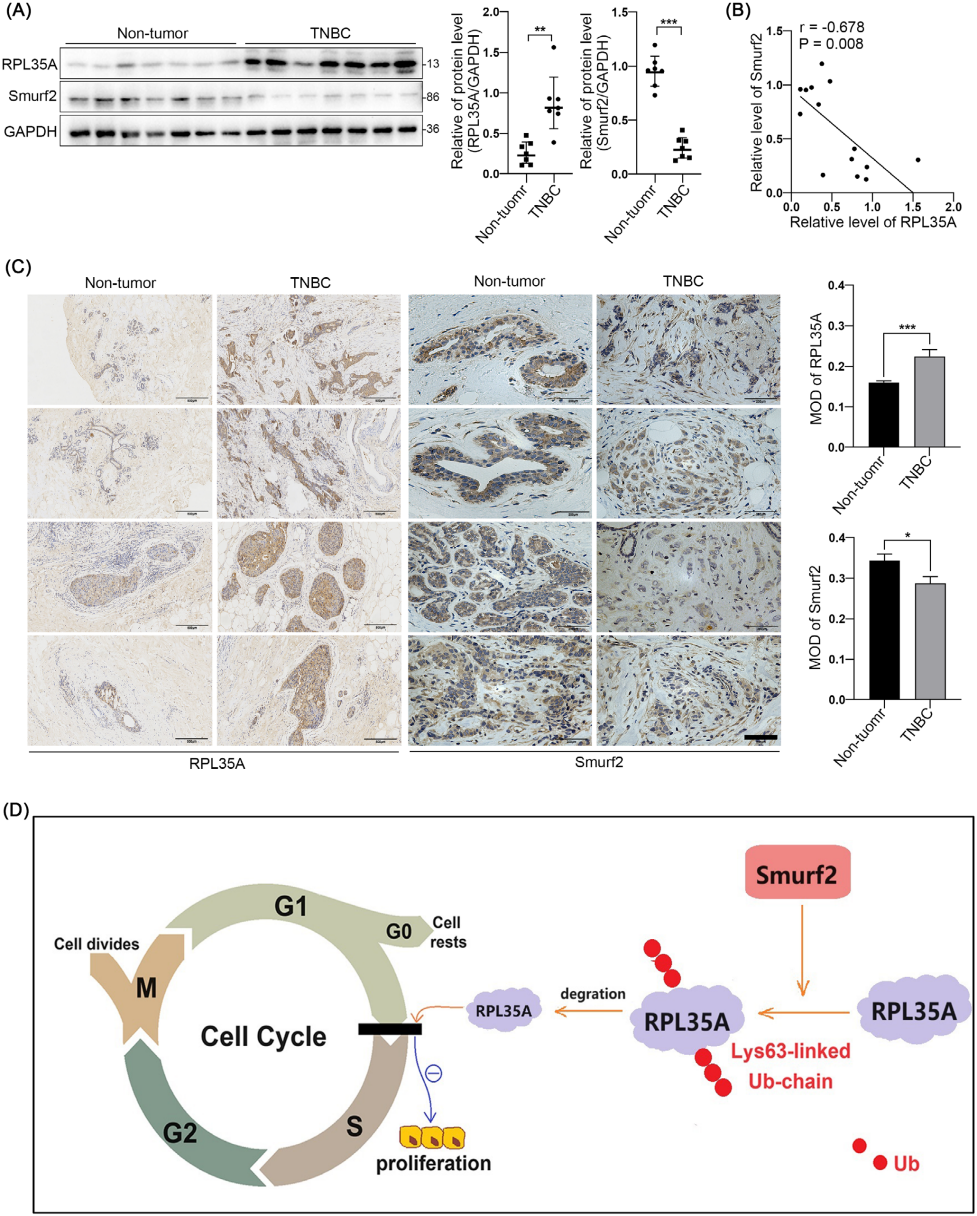
Smurf2 expression is inversely correlated with RPL35A expression in human TNBC tissues. (A) Protein levels of Smurf2 and RPL35A in 7 TNBC tissues and 7 paracancerous tissues. (B) Correlation of Smurf2 and RPL35A expression in TNBC tissues and paracancerous tissues. *r* = −0.678, *p* = 0.008. (C) Representative images of IHC staining of 4 paracancerous tissues versus 4 TNBC tissues, scale bar: 200 μm. (D) Schematic illustration of this study. *p < 0.05, **p < 0.01, ***p < 0.001.

As previously detailed, our findings indicate that Smurf2 downregulates RPL35A protein levels in TNBC cells via the proteasome pathway, thereby establishing a foundational regulatory relationship between these entities at the cellular level. As a result, we undertook a comprehensive investigation into the clinical implications of Smurf2 and RPL35A. It was showed that, in TNBC tissues, RPL35A expression was significantly higher than in adjacent non‐cancerous tissues, while Smurf2 protein levels were markedly lower compared to paracancerous tissues (Figure 6A), indicating that the expression levels of Smurf2 and RPL35A in human TNBC tissues were opposite. Further analysis of the correlation between RPL35A and Smurf2 revealed that the expression levels of RPL35A and Smurf2 were directly negatively correlated in both TNBC tissues and paraneoplastic tissues (*r* = −0.678, *p* = 0.008; Figure 6B). In addition, immunohistochemical analysis showed a significant increase in the mean optical density of RPL35A in TNBC tissues compared to adjacent non‐cancerous tissues, while Smurf2's mean optical density was notably decreased in TNBC relative to paracancerous tissues (Figure 6C). These results suggest that RPL35A expression in human TNBC tissues is negatively correlated with Smurf2.

## 4 Discussion

The traditional molecular classification of breast cancer includes luminal A, luminal B, HER2‐positive, and TNBC, with the prognosis for TNBC frequently being poor due to a limited availability of therapeutic targets [29, 30]. Therefore, there is an urgent necessity to identify more effective targets and novel therapeutic agents. In this study, we demonstrate that RPL35A acts as a tumour promoter in TNBC, suggesting its potential as a promising new therapeutic target.

RPL35A is identified as a pro‐carcinogenic factor, demonstrating increased expression in multiple tumour types [31, 32]. Recent studies have demonstrated that the deletion or mutation of RPL35A is correlated with diminished proliferation and increased apoptosis in haematopoietic cell lines, thereby contributing to Diamond‐Blackfan anaemia, which is commonly associated with dysregulated processing of ribosomal large subunit proteins [33, 34]. In addition, RPL35A has been reported as a tumour angiogenesis marker [35]. RPL35A could also interact with plague virus N‐terminal protease [36] and eukaryotic translation elongation factor 2 [37]. In this study, we elucidated the potential tumorigenic role of RPL35A in triple‐negative breast cancer (TNBC) and explored the underlying mechanisms. Our findings demonstrated that elevated expression levels of RPL35A in TNBC tissues were associated with a poor prognosis for patients. Functionally, silencing RPL35A led to the inhibition of TNBC cell proliferation and disruption of cell cycle progression.

The UPS constitutes a pivotal mechanism for the degradation of intracellular proteins, playing an essential role in preserving normal cellular function and maintaining homeostasis within the internal milieu [38]. We also demonstrated that RPL35A is degraded via the proteasome pathway. The function of UPS mainly depends on a set of highly coordinated cell enzymes, including E1 ubiquitin‐activating enzyme, E2 ubiquitin‐conjugating enzyme, and E3 ubiquitin‐ligase enzyme [39]. To identify the upstream ubiquitin ligases that modulate RPL35A, we employed the UbiBrowser and GeneCards databases to systematically screen for potential upstream ubiquitin ligases and interacting proteins. The findings suggest that SUMRF2 promotes the ubiquitination and subsequent degradation of RPL35A. Smurf2 is a member of the NEDD4 E3 ligase family. It has been reported that Smurf2 is involved in a variety of biological processes, such as cell cycle, proliferation, apoptosis, and metastasis [40, 41, 42]. Recent studies have shown that Smurf2 plays a dual role in human cancers, depending on protein interactions, tumour type, and other unclear factors [43, 44, 45]. Here, we identified that the upregulation of Smurf2 functions as a tumour suppressor in TNBC. In addition, Smurf2 is an E3 ubiquitin ligase known for modulating various substrates through ubiquitination modification, such as CNKSR2 [46], SIRT [47] and TβRI [48]. In this study, we identified Smurf2 as a potential upstream E3 ubiquitin ligase for RPL35A. Our findings indicate that Smurf2 facilitates the polyubiquitination of RPL35A; however, the loss of Smurf2's ligase activity did not result in any observable effects on the ubiquitination and degradation of RPL35A. This implies that Smurf2 specifically functions as an E3 ligase for RPL35A. Furthermore, we demonstrated that Smurf2 can impede cell cycle progression by modulating RPL35A, which subsequently inhibits the proliferation of TNBC cells.

Ubiquitin can form multiple structures through its seven lysine residues (K6, K11, K27, K29, K33, K48 and K63) and is recognised by different proteins as a multifunctional signal [49, 50]. Among these, K48‐ and K63‐linked ubiquitin chains are extensively characterised in the literature. Previous studies have shown that substrates modified by the K48‐linked chain are the best characterised and direct the modified protein to the proteasome for degradation [51]. The K63‐linked chain can not only participate in ubiquitination modification of substrates, but can also provide nonproteolytic signals, including DNA repair, signal transduction, and endocytosis [52]. In the present study, we demonstrated that Smurf2‐mediated polyubiquitination of RPL35A is initiated via a K63‐linked ubiquitin chain. This discovery may provide a basis for future investigations into the ubiquitination mechanisms of classical proteins.

In summary, we identified RPL35A as a novel substrate for Smurf2 and elucidated its regulatory mechanism. We propose that Smurf2 overexpression may lead to the frequent downregulation of RPL35A via K63‐linked polyubiquitination, hindering the proliferation of TNBC cells by obstructing the G1/S phase transition in the cell cycle (Figure 6D). This study offers new insights into the molecular mechanisms governing RPL35A and potential targeted therapies for TNBC. However, further investigation is needed to determine if RPL35A has additional roles in TNBC and whether other proteins can modulate its expression.

## Author Contributions

**Siyu Wei:** data curation (equal), formal analysis (equal), methodology (equal), writing – original draft (equal). **Yuying Liu:** data curation (equal), formal analysis (equal), methodology (equal). **Zhihao Wang:** data curation (equal). **Ti Wei:** data curation (equal), investigation (equal). **Wenkai Zhou:** conceptualization (equal), resources (equal). **Wanwan Li:** conceptualization (equal), funding acquisition (equal), methodology (equal). **Jiaxin Zhang:** conceptualization (equal), writing – review and editing (equal). **Zhiyi Liu:** supervision (equal), validation (equal). **Zhao Liu:** supervision (equal), visualization (equal).

## Ethics Statement

The study was approved by the Ethics Committee of The Affiliated Hospital of Xuzhou Medical University (approval no. XYFY2022‐KL405‐01).

## Consent

Informed consent was obtained from patients for all samples.

## Conflicts of Interest

The authors declare no conflicts of interest.

## Data Availability

The data that support the findings of this study are available from the corresponding author upon reasonable request.
